# Molecular-Network Transformations in Tetra-Arsenic Triselenide Glassy Alloys Tuned within Nanomilling Platform

**DOI:** 10.3390/molecules29143245

**Published:** 2024-07-09

**Authors:** Oleh Shpotyuk, Malgorzata Hyla, Yaroslav Shpotyuk, Zdenka Lukáčová Bujňáková, Peter Baláž, Pavlo Demchenko, Andrzej Kozdraś, Vitaliy Boyko, Andriy Kovalskiy

**Affiliations:** 1 Institute of Physics, Jan Dlugosz University in Częstochowa, 13/15, al. Armii Krajowej, 42-200 Częstochowa, Poland; mhyla@ujd.edu.pl; 2 O.G. Vlokh Institute of Physical Optics, Ivan Franko National University of Lviv, 23, Dragomanov Str., 79005 Lviv, Ukraine; boykovit@gmail.com; 3 Department of Sensor and Semiconductor Electronics, Ivan Franko National University of Lviv, 107, Tarnavskoho Str., 79017 Lviv, Ukraine; yashpotyuk@gmail.com; 4 Institute of Physics, University of Rzeszow, 1, Pigonia Str., 35-959 Rzeszow, Poland; 5 Institute of Geotechnics of Slovak Academy of Sciences, 45, Watsonova Str., 04001 Košice, Slovakia; bujnakova@saske.sk (Z.L.B.); balaz@saske.sk (P.B.); 6 Department of Inorganic Chemistry, Ivan Franko National University of Lviv, 6, Kyryla i Mefodiya Str., 79000 Lviv, Ukraine; demchenko@lnu.edu.ua; 7 Faculty of Physics, Opole University of Technology, 75, Ozimska Str., 45-370 Opole, Poland; a.kozdras@po.opole.pl; 8 Department of Physics, Engineering and Astronomy, Austin Peay State University, Clarksville, TN 37044, USA; kovalskiya@apsu.edu

**Keywords:** characterization, modelling, thermal analysis, X-ray analysis, glass, powder methods, phase transformation

## Abstract

Polyamorphic transformations driven by high-energy mechanical ball milling (nanomilling) are recognized in a melt-quenched glassy alloy of tetra-arsenic triselenide (As_4_Se_3_). We employed XRPD analysis complemented by thermophysical heat-transfer and micro-Raman spectroscopy studies. A straightforward interpretation of the medium-range structural response to milling-driven reamorphization is developed within a modified microcrystalline model by treating diffuse peak-halos in the XRPD patterns of this alloy as a superposition of the Bragg-diffraction contribution from inter-planar correlations, which are supplemented by the Ehrenfest-diffraction contribution from inter-atomic and/or inter-molecular correlations related to derivatives of thioarsenide As_4_Se_n_ molecules, mainly dimorphite-type As_4_Se_3_ ones. These cage molecules are merely destroyed under milling, facilitating the formation of a polymerized network with enhanced calorimetric heat-transfer responses. Disruption of intermediate-range ordering, due to weakening of the FSDP (the first sharp diffraction peak), accompanied by an enhancement of extended-range ordering, due to fragmentation of structural entities responsible for the SSDP (the second sharp diffraction peak), occurs as an interplay between medium-range structural levels in the reamorphized As_4_Se_3_ glass alloy. Nanomilling-driven destruction of thioarsenide As_4_Se_n_ molecules followed by incorporation of their remnants into a glassy network is proved by micro-Raman spectroscopy. Microstructure scenarios of the molecular-to-network polyamorphic transformations caused by the decomposition of the As_4_Se_3_ molecules and their direct destruction under grinding are recognized by an ab initio quantum-chemical cluster-modeling algorithm.

## 1. Introduction

Nowadays, the nm-scaled substances functionalized through a plethora of nanostructurization technologies, such as high-energy mechanical milling (MM, also referred to as *nanomilling*), compose one of the most promising challenges in contemporary nanomaterials science and engineering [1]. Employing mechanochemistry [2,3], the thermodynamically stable materials can be transformed into out-of-equilibrium high-entropy prototypes, with this transition being simply observable in crystals possessing regular interatomic ordering, while merely hidden in amorphous substances, such as glasses derived by conventional melt-quenching (MQ). Nevertheless, even in the latter case, many glassy materials are modified irreversibly because of the MM-driven nanostructurization metastability [4].

In past decades, this conceptual approach has been convincingly proved for MQ-derived substances, like chalcogenide glasses (ChG) [5,6], with their archetypal representatives from a binary As-Se system (hereafter referred to as glassy arsenoselenides g-As_x_Se_100−x_) possessing a great variety of molecular-network conformations in a whole glass-forming region from ‘pure’ Se (*x* = 0) to As-rich alloys with *x*~65–75 [5,6,7]. The stoichiometric arsenic triselenide As_2_Se_3_ has a characteristically layered network composed of corner-sharing trigonal AsSe_3/2_ pyramids interlinked by -Se- bridges, which can be classified as optimal in view of the average number of mechanical constraints per atom *n_c_* exactly approaching space dimensionality (3D), representing the principal glass-former in As-Se systems. In this stoichiometric glass-former and under-stoichiometric Se-bearing g-As_x_Se_100−x_ alloys from moderated compositional domains (20 < *x* < 40) dominated by the transition from layer-type structures characteristic of As_2_Se_3_ to a 2D-network of Se-chains bridging AsSe_3/2_ pyramids [3], the effects of nanomilling-driven nanostructurization are merely hidden [8,9]. With a trend further towards ‘pure’ Se, that is, under-stoichiometric g-As_x_Se_100−x_ enriched in Se content (x < ~10–15), where the molecular Se_8_ ring-like species consist of *cis*-configurated Se linkages typical for α- and β-monoclinic Se are stabilized, in addition to spiral *trans*-configurated Se_n_ chains typical for trigonal t-Se [10], nanomilling induces molecular-to-network reamorphization transformations of Se chains bridging AsSe_3/2_ pyramids from preferential *cis-* to *trans*-configurated arrangements [11]. Especially attractive for the glass manufacturing community is the recently justified possibility to modify g-As_x_Se_100−x_ in over-stoichiometric compositional domain (*x* > 40), tuning them by cage-like molecules such as As_4_Se_4_, As_4_Se_3_ or As_4_ incorporated in covalent-bonded As-Se networks [3]. In these over-stoichiometric As-bearing arsenoselenides, nanomilling-driven escape from the macro- to the nanoscopic state is expected for tetra-arsenic selenide compounds having stable crystalline counterparts (alternatively, *thioarsenides* As_4_Se_n_ where *n =* 4, 3) [1,3,9]. Recently, the transition from the initial to final amorphous states (that can be considered as a manifestation of amorphous-I-to-amorphous-II or reamorphization transition) has been realized under nanomilling in MQ-derived arsenic monoselenide, g-AsSe (*viz*. tetra-arsenic tetraselenide, g-As_4_Se_4_), contributing to considerable progress in the engineering of special glass media with guided functionality [12].

The objective of this research is to justify the molecular-to-network nature of the *na*nomilling-driven polyamorphic transition in glassy arsenoselenides g-As_x_Se_100−x_ of other remarkable compositions equivalent to tetra-arsenic triselenide, such as g-As_4_Se_3_ (corresponding to *x* = 57, g-As_57_Se_43_), having the orthorhombic As_4_Se_3_ as the high-temperature crystalline counterpart [13,14,15]. The microstructure of the MQ-derived and nanomilled specimens will be recognized by employing X-ray powder diffraction (XRPD) analysis in application to diffuse peak-halos responsible for medium-range ordering in ChG. This study on molecular-network disproportionality in arsenoselenide alloys compositionally approaching As_4_Se_3_ will be complemented with calorimetric heat-transfer measurements and micro-Raman scattering (micro-RS) spectroscopy studies, and ab initio quantum-chemical modeling of thioarsenide As_4_Se_n_ molecules and their network-forming derivatives using the cluster-simulation code CINCA (the cation-interlinked network cluster approach [16,17]).

## 2. Results and Discussion

### 2.1. Medium-Range Structural Correlations in MQ-Derived g-As_4_Se_3_

**Figure 1:**
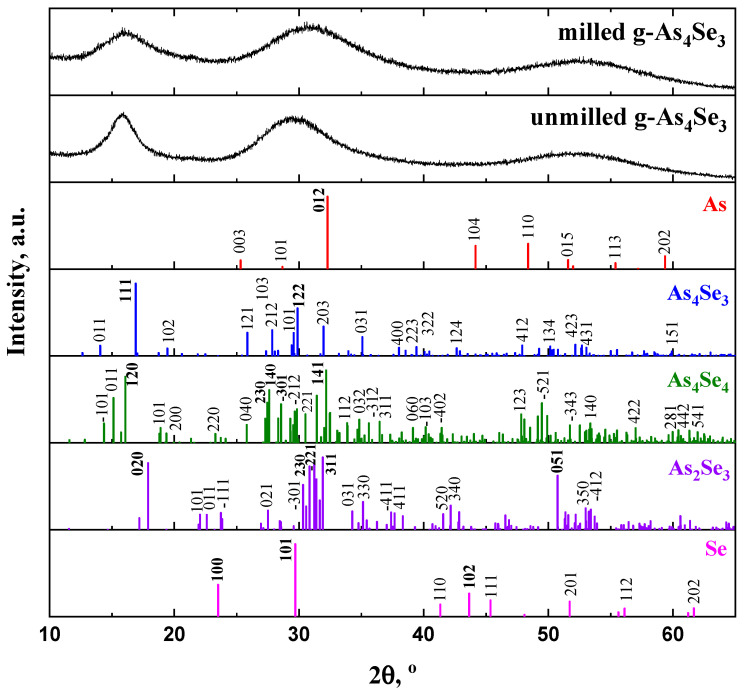
The XRPD patterns of unmilled MQ-derived and dry-milled g-As_57_Se_43_ alloys showing regions of the three most prominent diffuse peak-halos corresponding to the FSDP (~15–25°2*θ*), SSDP (~28–33°2*θ*) and TDP (~50–60°2*θ*). Theoretical Bragg-diffraction reflexes of monoclinic As_2_Se_3_ (JCPDS No. 65-2365) [27,28], monoclinic As_4_Se_4_ (JCPDS No. 71-0388), orthorhombic As_4_Se_3_ (JCPDS No. 04-4979), trigonal Se (JCPDS No. 73-0465) and rhombohedral As (JCPDS No. 72-1048) are reproduced below for comparison (see text for more details).

The XRPD patterns collected for MQ-derived g-As_57_Se_43_ before and after high-energy MM in a dry mode are depicted in Figure 1.

These patterns clearly demonstrate a so-called three-peak structure [18] composed of separated peak-halos responsible for the first sharp diffraction peak (FSDP), second sharp diffraction peak (SSDP) and third diffraction peak (TDP), supplemented by some features related to pre-FSDP, post-FSDP and post-SSDP (unreproducible in the reduced structure factor determination) positioned near diffraction angles 2*θ* and scattering vectors *Q* character for g-As_60_Se_40_ [9]. Thus, the FSDP position in the MQ-derived g-As_4_Se_3_ (*viz*. characteristic distance *R*~5.7 Å) is found to be in excellent agreement with the most pronounced Bragg-diffraction line (*I* = 100%) arising from (111) plane in the orthorhombic As_4_Se_3_ (*equiv*. to inter-planar distance *d* = 5.243 Å) [13]. As it follows from Figure 1, one of the strongest (*I* = 91.3%) lines arising from the (120) plane at ~16.07°2*θ* (*d* = 5.512 Å) in other molecular thioarsenide (monoclinic As_4_Se_4_ [19,20,21]) occurs to be very close to the FSDP, although the other line (*I* = 91.2%) ascribed to (020) plane in layer-type monoclinic As_2_Se_3_ [19,22] is positioned at higher angle (~17.9°2*θ*) corresponding to *d* = 4.950 Å. Assuming equal contributions to the FSDP from these lines (ascribed to As_4_Se_3_-, As_4_Se_4_- and As_2_Se_3_-structures), the FSDP-related characteristic distance *R* was expected near ~5.64 Å, which is slightly below the characteristic value derived from the Bragg-diffraction positioning of the FSDP. This testifies in favor of the essential contribution to the FSDP from inter-planar correlations belonging to some remnants of crystalline arsenoselenide structures.

Other input to the FSDP is expected from inter-atomic and/or inter-molecular correlations, which belong to these remnants. Indeed, as was pointed out in [5,6,7], the structure of over-stoichiometric As-rich g-As_x_Se_100−x_ (*x* > 40) could be imagined as a stacking of network-type entities based on Se-linked AsSe_3/2_ pyramids and molecular-type entities based on thioarsenide cages (such as As_4_Se_4_, As_4_Se_3_, and even As_4_). The spatial arrangement of such cages could be parameterized by introducing a ‘dummy atom’ B serving as a geometrical barycentre for each molecule [23]. The dense random packing of such thioarsenide molecules (*viz*. inter-molecular correlations) contributes to diffuse peak-halos in the XRPD patterning of arsenoselenides through the Ehrenfest diffraction [24,25,26,27]. 

**Figure 2:**
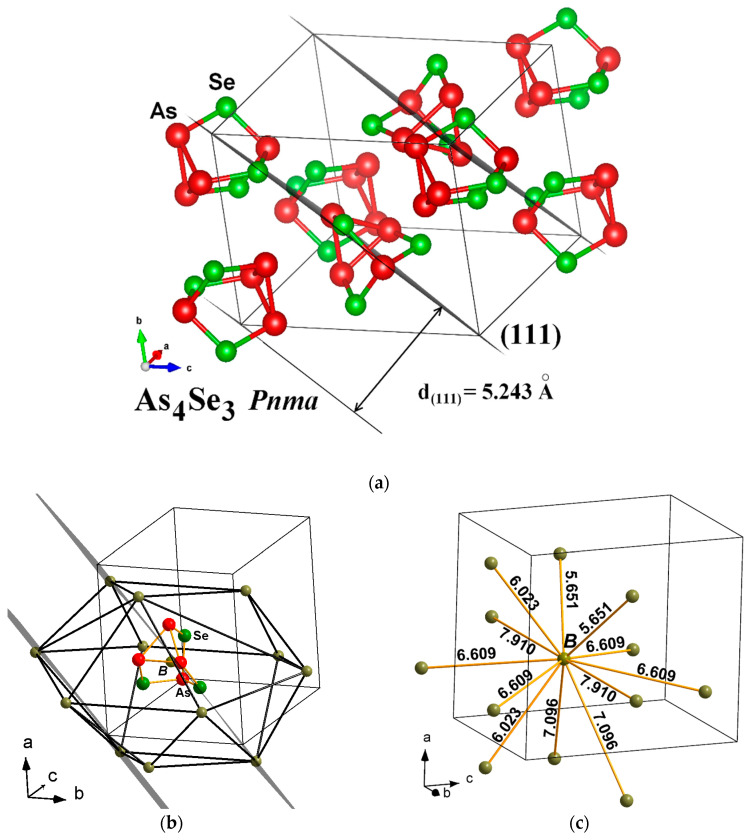
The reconstructed structural fragment of orthorhombic As_4_Se_3_ showing (**a**)—arrangement of cage-like As_4_Se_3_ molecules in respect to the family of (111) planes corresponding to the strongest Bragg-diffraction line, (**b**)—As_4_Se_3_ molecule (centered in B) in surrounding of 12 neighbors forming B[B_12_] anticubooctahedron; (**c**)—possible inter-molecular centroid-centroid distances B-B within B[B_12_] polyhedron (in Å). The averaged B-B distance around each ‘dummy atom’ derived from hexagonal close packing of 12 molecules d_B-B_(As_4_Se_3_) approaches 6.650 Ǻ (see text for more details).

A typical fragment of orthorhombic As_4_Se_3_ structure visualized from crystallographic data of Bastow and Whitfied [13] using the DIAMOND and VESTA programs is shown in Figure 2. The arrangement of As_4_Se_3_ molecules is reproduced with respect to the strongest Bragg-diffraction line arising from the (111) plane in the orthorhombic As_4_Se_3_, with an inter-planar distance *d* = 5.243 Å (Figure 2a). Each As_4_Se_3_ molecule is surrounded by 12 neighbors forming B[B_12_] anticubooctahedron, corresponding to hexagonal close packing of a Mg structure type (see Figure 2b), with inter-molecular centroid-centroid distances deviated from 5.651 Ǻ to 7.910 Ǻ (Figure 2c), corresponding in average to d_B-B_ = ~6.650 Ǻ. This distance, accepted as the radius of the first coordination sphere in the dense packing of As_4_Se_3_ cage-like molecules obeying the Ehrenfest relation [27], corresponds to the FSDP position in the MQ-derived (unmilled) g-As_4_Se_3_ approaching *d_s_*~7.0 Å (see Figure 1).

Thus, the FSDP in g-As_4_Se_3_ at *Q*_1_ = *Q_FSDP_*~1.11 Ǻ^−1^ can be attributed to equal contributions from both *inter-planar* correlations corresponding to some remnants ascribed to As_4_Se_3_-, As_4_Se_4_- and As_2_Se_3_-type crystalline structures with an averaged Bragg-diffraction distance *R* = ~5.7 Å and respective *inter-molecular* correlations with averaged Ehrenfest-diffraction distance *d_s_*~7.0 Å.

Noteworthy, because of the Ehrenfest diffraction due to pair inter-atomic correlations within some remnants of crystalline structures, the diffuse peak-halos in the XRPD patterning of ChG reveal non-elementary satellite nature supplemented by some humps and asymmetric extensions [9,12]. Thus, in g-As_4_Se_3_, the shoulder near ~1.5 Ǻ^−1^ (*viz*. *d_s_*~5.1 Å), referred to as the post-FSDP, is revealed at the high-angular side of the FSDP, so that both peak positions obey interrelation:

*κ*(*FSDP*) = *Q*_*post*-*FSDP*_/*Q*_*FSDP*_ = *d*_*s*_^*FSDP*^/*d*_*s*_^*post*-*FSDP*^ = 1.38, (1)

which occurs very close to the Ehrenfest number (1.23) [27].

In a similar manner, it is found that a slight hump near ~2.4 Ǻ^−1^ (*viz*. *d_s_*~3.2 Å) appears as satellite doublet to the SSDP, justifying its asymmetry (reasonably referred to as the post-SSDP), with both peaks obeying similar interrelation, which is also very close to the Ehrenfest number [27]:

*κ*(*SSDP*) = *Q*_*post*-*SSDP*_/*Q*_*SSDP*_ = *d*_*s*_^*SSDP*^/*d*_*s*_^*post*-*SSDP*^ = 1.15. (2)

Thus, the asymmetry observed in both peak-halos (the FSDP and the SSDP) is presumably caused by the superposition of the broadened Bragg-diffraction reflections from remnants of quasi-crystalline inter-planar correlations superimposed by the Ehrenfest-diffraction reflections from the most prominent inter-atomic and/or inter-molecular correlations belonging to these remnants. In contrast, the TDP at the higher diffraction angles, corresponding to *Q*_3_ = *Q_TDP_*~3.6 Å^−1^, associated with direct nearest-neighbor correlations approaching *d_s_*~2.1 Ǻ, does not show any *doublet* structure.

The Ehrenfest diffraction is a suitable approach to explain other features in the XRPD patterning of ChG, known as pre-FSDP [28] and related to an additional peak-halo appearing at ~(5–7)°2*θ*, i.e., in the region free of any inter-planar reflections from all possible crystalline counterparts. This peak-halo (unreproducible with respect to the structure factor determination and compositional variations [9,12]) arises from prolonged *inter-atomic correlations* in g-As_4_Se_3_ approaching *d_s_*~16.5 Ǻ.

### 2.2. Medium-Range Structure Response in g-As_4_Se_3_ on Nanomilling-Driven Reamorphization

As emerged from Figure 1, high-energy MM does not alter the principal appearance of diffuse peak-halos in the XRPD pattern of g-As_57_Se_43_, testifying in favor of a nanomilling-driven polyamorphic transition between respective states of unmilled and milled alloys. Following Propenzi et al. [29], this effect can be classified as MM-induced polyamorphism, whereas the transition between respective states of glass before and after MM can be referred to as nanomilling-driven *reamorphization* transition [12]. 

Changes observed in diffuse peak-halos in g-As_4_Se_3_ undergoing nanomilling-driven reamorphization are well understood, even from the visual inspection of Figure 1. Indeed, after MM, the FSDP loses intensity and gets to be (i) more weakened, (ii) shifted to higher *Q*_1_ = *Q_FSDP_*~1.14Ǻ^−1^ and (iii) broadened in width to Δ*Q*_1_ = Δ*Q_FSDP_*~0.32 Ǻ^−1^. Therefore, the spacing of the FSDP-responsible quasi-periodicity *R* in g-As_3_Se_4_ slightly decreases after MM to ~5.5 Ǻ, while correlation length *L* (over which this quasi-periodicity is maintained) gradually decreases to ~19.5 Ǻ. This means the MM fragmentation impact that occurs in the correlation length *L* of the quasi-periodic entities is responsible for the FSDP. 

Similar, albeit reduced, changes are observed in the SSDP (increase in the position of this peak-halo, Q_2_ = Q_SSDP_, broadening in the width and ΔQ_2_ = ΔQ_SSDP_), signifying the nanomilling-driven fragmentation of the correlation length of the structural entities is responsible for this peak-halo. Since the XRPD analysis is arranged following the normalization procedure with respect to the maximum peak, these changes should be accepted as a signature of increased ERO.

In contrast, as can be also inferred from previous research [8,9,12], no changes were found in the TDP, presumably due to invariant nearest-neighbor interatomic correlations in these arsenoselenides. 

Thus, disruption of IRO due to weakening of the FSDP (when the FSDP loses intensity, becomes more broadened in width and shifted towards higher diffraction angles, as follows from Figure 1) is accompanied by an enhancement of ERO due to fragmentation of the SSDP responsible entities (when the SSDP becomes broadened and shifted towards higher angles; this also follows from Figure 1). This occurs as a manifestation of the interplay between IRO and ERO in the examined molecular-network glassy alloy (g-As_3_Se_4_) undergoing nanomilling-driven *reamorphization*. Ultimately, structural correlations responsible for IRO (presumably of inter-molecular and inter-atomic nature) are substantially destroyed under MM at a cost of competitive inter-planar quasi-crystalline correlations, taking the nanomilled glassy arsenoselenides closer to layer-type stoichiometric g-As_2_Se_3_ [8,15].

A similar competition at different levels of medium-range structural organization associated with IRO and ERO is characteristic of pressure-induced polyamorphism in glassy substances [30,31,32,33]. Thus, under applied pressure to molecular-network ChG [30,32,33], changes in IRO dominate, and inter-molecular spacing decreases because of increased packing of molecular units (causing gradual densification of a glass). As a result, the FSDP shifts to higher *Q*_1_ and reduces dramatically until completely disappearing at higher pressures (~10–20 GPa), signalizing IRO breaking or collapse. The weakening of the FSDP under pressurization is accompanied by a growth in the SSDP, and its shift to higher *Q*_2_ is indicative of an ERO increase. Despite FSDP and SSDP being restored under pressure release, changes in these halos serve as a signature of elementary pressure-induced IRO-to-ERO interplay, which also occurs in nanomilled molecular-network ChG like g-AsSe [12] or g-As_3_Se_4_. So high-energy MM (as in this case) is an effective way to stabilize such changes in molecular-network glass as a result of nanomilling-driven *irreversible* reamorphization.

The doublet structure of the FSDP, revealed through satellite high-angular post-FSDP, is more expressed after MM because *κ*(FSDP)~1.34 in Equation (1) better approaches the Ehrenfest number (1.23) [27]. It means that *inter-atomic* correlations contributing to the FSDP (*d_s_^FSDP^*) prevail over *inter-atomic* correlations connected with *inter-planar* arrangement responsible for this peak-halo (*d_s_^post-FSDP^*). Concisely, disruption of intermediate-range ordering in the glasses subjected to nanomilling is dominated as compared with the destruction of inter-atomic correlations belonging to remnants of quasi-crystalline planes contributing to the FSDP. So, it seems reasonable that asymmetry in the FSDP is reduced after MM (Figure 1). In contrast, the doublet structure of the SSDP revealed in the satellite post-SSDP is depressed after MM, which resulted in *κ*(*SSDP*) approaching only ~1.12.

Thereby, remnants of crystalline entities in g-As_4_Se_3_, responsible for *inter-molecular* correlations with inter-centroid distances between As_4_Se_3_ cages d_B-B_(α-As_4_Se_3_)~6.650 Ǻ contributing to the FSDP through the Ehrenfest diffraction, are destroyed under MM, similarly to more depressed *inter-planar* quasi-crystalline correlations contributing to the FSDP through the Bragg-diffraction lines ascribed to the (111) plane in orthorhombic As_4_Se_3_ (I = 100%), the (120) plane in monoclinic As_4_Se_4_ (*I* = 91.3%) and the (020) plane in monoclinic As_2_Se_3_ (*I* = 91.2%). As a result, the FSDP in g-As_4_Se_3_, obeying such molecular-to-network reamorphization*,* becomes gradually weakened in intensity, broadened in width and shifted towards higher scattering vector positions (see Figure 1).

Noteworthy, changes in medium-range structure in the examined molecular-network glassy alloy (g-As_4_Se_3_) associated with nanomilling-driven reamorphization obey clearly an irreversible scenario. This is in contrast to the pressure-induced *reversible* amorphous-to-amorphous transition observed by Ahhmad et al. [31] in network glasses (like g-As_2_Se_3_) under compression-decompression cycling below ~40 GPa, which became amorphous-to-crystalline (also reversible) as the pressure was increased to ~53.5 GPa. However, XRD patterns in [31] were not parameterized, leaving room to consider that this was only based on visual inspection of XRD profiles for glasses before and after compression. Thus, it was obvious that under the increasing pressure (up to ~13.5 GPa) applied to g-As_2_Se_3_, changes in peak-halos were similar and commensurable to those observed in MM-driven reamorphization [8,9,12], while after decompression to the initial state, a gradual difference still remained.

### 2.3. Thermophysical Heat-Transfer Phenomena and Micro-RS Response in g-As_4_Se_3_ Undergoing Nanomilling-Driven Molecular-to-Network Transition

**Figure 3:**
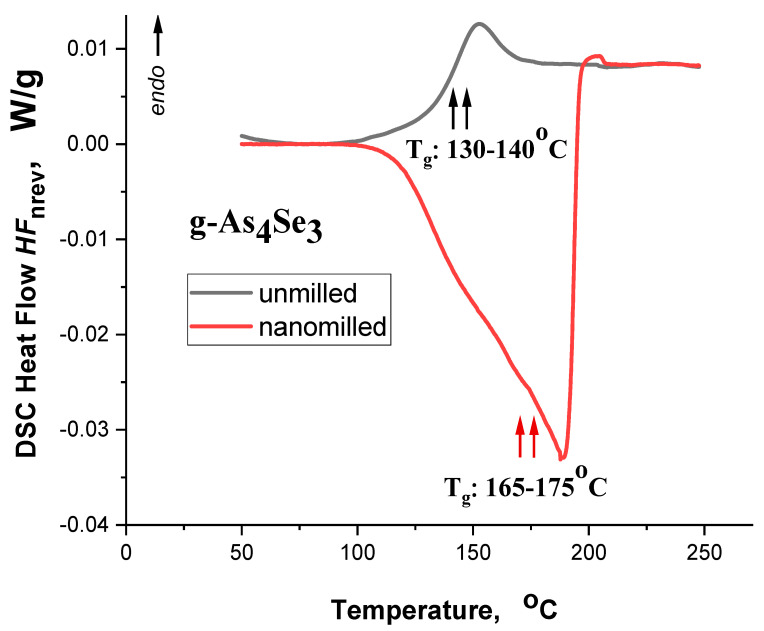
The hf-DSC thermograms detected in the dynamic heating-run regime show the variations in non-reversing DSC heat flow *HF*_nrev_ in unmilled (black curve) and nanomilled (red curve) As_4_Se_3_. The glass transition temperature *T*_g_ enhancement in this molecular-network glassy alloy undergoing nanomilling-driven amorphous-I-to-amorphous-II (reamorphization) transition is obvious.

Temperature variations in non-reversing heat flow *HF*_nrev_ in g-As_4_Se_3_ in initial (melt-quenched) and final (reamorphized) states in MQ-derived alloy subjected to nanomilling collected in the dynamic heating-run regime are reproduced by, respectively, the colored black and red thermograms in Figure 3. Under heating, the principal calorimetric heat-transfer event in these alloys derived by MQ represents a glass transition [34,35]. In molecular-network g-As_4_Se_3_, this thermal-alteration event is revealed as a sharp *endothermic* step-like jump near ~130–140 °C in temperature behavior of non-reversing heat flow *HF*_nrev_ depicted by the black curve in Figure 3. More precise parameterization of this phenomenon using the multifrequency DSC-TOPEM^®^ method for As-Se alloys of close composition such as g-As_55_Se_45_ [35] provides the values of the onset of the glass transition temperature *T*_g_ ~134.6 °C, heat capacity variation Δ*C*_p_~0.11 J·g^−1^·K^−1^ and specific enthalpies difference Δ*H*~5.57 J·g^−1^.

This calorimetric response originated from temperature variation in DSC heat flow *HF*_nrev_ gets to be changed drastically in g-As_4_Se_3_ subjected to nanomilling, this effect being revealed as a pronounced *exothermic* event within broad 100–200 °C region highlighted by the red curve in Figure 3. Undoubtedly, this phenomenon is due to the relaxation of inner strength generated in the examined glass under high-energy MM. Slight features observed in *HF*_nrev_ variation near 165–175 °C testify in favor of enhanced glass transition temperature in this glass undergoing MM-driven molecular-to-network transition, in contrast to *T*_g_ reduction observed in network glasses such as g-As_5_Se_95_ [35]. Parameterization of this calorimetric response for g-As_55_Se_45_ specified with DSC-TOPEM^®^ method shows the onset glass-transition temperature *T*_g_ increased to ~170.0 °C, Δ*C*_p_~0.13 J·g^−1^·K^−1^ and Δ*H*~−29.7 J·g^−1^ [35]. 

**Figure 4:**
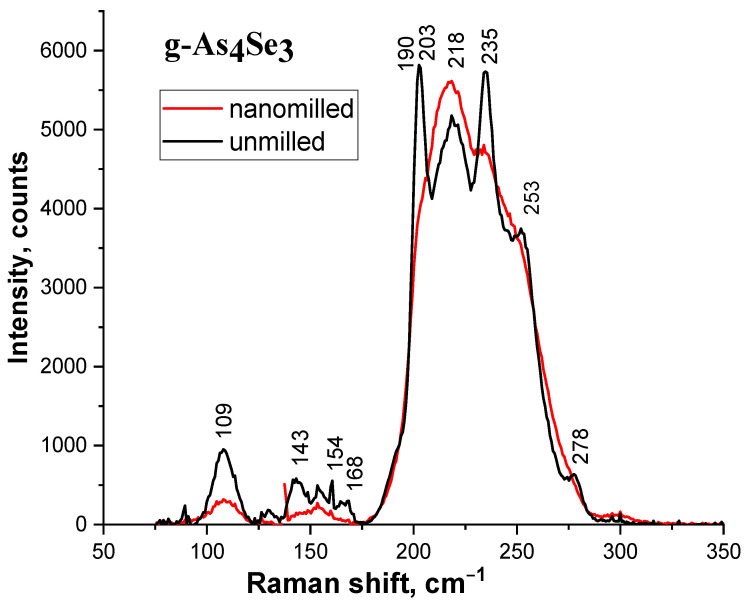
The micro-RS spectra collected from unmilled (black curve) and nanomilled (red curve) samples of g-As_4_Se_3_ (the most prominent RS-active bands in unmilled glass alloy are distinguished).

The network directionality in reamorphization is also confirmed by micro-RS response observed in MQ-derived g-As_4_Se_3_ subjected to MM, with respective micro-RS spectra being reproduced in Figure 4. The most prominent RS-active modes in a non-milled sample are represented by a few more or less resolved low-frequency bands (at ~109, 143, 154, 168, 190 cm^−1^) and high-frequency bands (at ~203, 218, 235, 253, 278 cm^−1^). The high-frequency bands (strong and very strong) are ascribed to overlapped bond-stretching modes of AsSe_3/2_ pyramids (227 cm^−1^ [35,36,37]) and cage-like thioarsenide molecules, such as As_4_Se_4_ (190 and 248 cm^−1^ [38]), As_4_Se_3_ (196, 242, 256, 266 and 280 cm^−1^ [39]) and As_4_ (~200 cm^−1^ [40]). The low-frequency bands (preferentially weak and medium) are ascribed to bond-bending modes of molecular cages, such as As_4_Se_4_ (106, 136, 144, 190, 207 cm^−1^ [40,41]) and As_4_Se_3_ (140 and 166 cm^−1^ [39]). These features are merely broadened and depressed in g-As_4_Se_3_ subjected to nanomilling (see Figure 4, red curve), testifying in favor of nanomilling-driven destruction of thioarsenide As_4_Se_n_ molecules and their incorporation in newly polymerized As-Se backbone. 

Thus, the structure of g-As_4_Se_3_ becomes notably stressed under MM being affected by defects. As a result, the remainder of destroyed thioarsenide molecules became more incorporated in a network, changing essentially calorimetric heat-transfer response from the glass-transition event in this alloy.

### 2.4. Cluster Modeling of Molecular-Network Conformations Related to As_4_Se_3_ Thioarsenide

**Figure 5:**
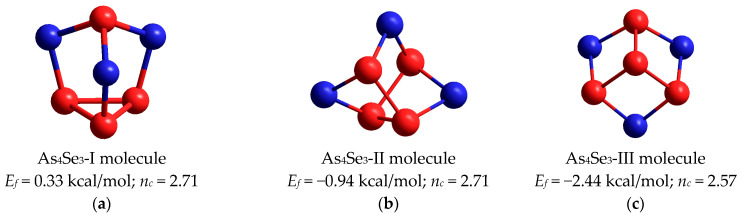
The optimized ball-and-stick presentation of three types of tetra-arsenic triselenide (As_4_Se_3_) cage molecules possessing different arrangement of neighboring As atoms: (**a**)—dimorphite-type As_4_Se_3_-I in *triangular-pyramidal* (As_3_)-As configuration; (**b**)—As_4_Se_3_-II in *chain*-like (*zig-zag*) As4 configuration; (**c**)—As_4_Se_3_-III in *star*-like As(As)3 configuration. The Se and As atoms are blue- and red-colored, and bonds between these atoms are denoted by respective colored sticks. The cluster-forming energies *E_f_* are determined with respect to the energy of a single AsSe_3/2_ pyramid.

There are three conformations for tetra-arsenic triselenide molecule (As_4_Se_3_) in dependence on the arrangement of four As atoms, forming three (As-As) bonds. These include *triangular-pyramidal* (As_3_)-As conformation due to basal (As_3_) triangular neighboring with AsSe_3_ pyramid by sharing three -Se- bridges along adjacent apical edges (see Figure 5a), open *chain*-like As4 conformation due to three (As-As) bonds in *zig-zag* sequence of As atoms (Figure 5b) and *star*-like As(As)3 conformation due to three (As-As) bonds having the same origin on As atom (Figure 5c).

In view of average cluster-forming energies (*E_f_*) calculated for these conformations employing the CINCA algorithm [16,17], the most plausible is As_4_Se_3_-I molecule composing *triangular-pyramidal* (As_3_)-As arrangement (shown in Figure 5a) possessing *E_f_* = 0.33 kcal/mol with respect to the energy of single AsSe_3/2_ pyramid [17]. This cage-like molecular cluster is isostructural with the molecule refined in α- and β-modifications of mineral dimorphite As_4_S_3_ by Whitfield [23,41,42,43]. Topologically, this molecule includes four small rings (three pentagons and one triangle) in idealized under-constrained geometry of *C_3v_* symmetry because of the average number of constraints *n_c_* = 2.71, which is evidently less than space dimensionality (3D). All seven atoms of almost spherical shape are positioned at the same sphere, resulting in a 0D structure with unusually low calorimetric heat transfer and strong thermal expansion responses, which are characteristic features of the plastically crystalline As_4_Se_3_ phase [15,30]. With respect to the CINCA modeling, the optimized configuration of this As_4_Se_3_-I molecule is defined by slightly deviated (As-Se) and (As-As) bond lengths approaching 2.37 Å and 2.46 Å, respectively, and bond angles on apical As atoms ∠(Se-As-Se) = 99.4°, on basal As atoms in linking to AsSe_3_ pyramid ∠(As-As-Se) = 104.1°, on basal As atoms within (As_3_) triangular ∠(As-As-As) = 60.0°, and angles of -Se- bridges between apical and basal As atoms ∠(As-Se-As) = 101.9°. 

Other molecular clusters corresponding to As_4_Se_3_ stoichiometry are unfavorable as compared with this dimorphite-type As_4_Se_3_-I molecule. Thus, the estimated *E_f_* energy approaches −0.94 kcal/mol for the As_4_Se_3_-II molecule arranged in *chain*-like As4 configuration (as shown in Figure 5b) and −2.44 kcal/mol for the As_4_Se_3_-III molecule arranged in *star*-like As(As)3 configuration (as shown in Figure 5c), both molecules being under-constrained in view of the large number of small rings involved. 

Thus, in MQ-derived glasses compositionally approaching As_4_Se_3_, the molecular clusters of the first type (dimorphite-type As_4_Se_3_-I molecule) are obviously dominant. But not only these clusters are governing in these alloys. As it follows from the above results of calorimetric measurements and micro-RS studies, the examined glassy arsenoselenides are also enriched in realgar-type As_4_Se_4_ molecules and amorphous As-bearing network substance of unidentified composition obeying the concept of preferential molecular-network disproportionality [44,45]. By analogy with As-S alloys [45], these products are expected in the MQ-derived As-Se alloys near As_4_Se_3_ stoichiometry, as a result of the decomposition of most energetically favorable As_4_Se_3_-I cage-like molecules. 

In glassy-crystalline As-S alloys, where multiphase equilibria are disturbed by the transformation of dimorphite As_4_S_3_ phase into realgar-type β-As_4_S_4_ phase [15], the supplemented amorphous substance is compositionally close to a-As_4_S_2_ and decomposition reaction is activated under high energetic barrier Δ*E_f_*~1.5 kcal/mol [44]:

2·(As_4_S_3_-I) → β-As_4_S_4_ + a-As_4_S_2_ + (Δ*E_f_* = +1.53 kcal/mol). (3)

Let us parameterize the decomposition reaction in glassy arsenoselenide g-As_4_Se_3_ on the basis of cluster-forming energies *E_f_* calculated for the respective components.

**Figure 6:**
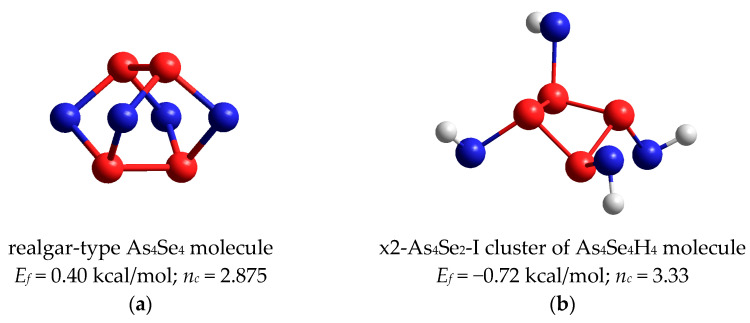
The ball-and-stick presentations of main products of decomposition reaction in g-As_4_Se_3_: (**a**)—realgar-type As_4_Se_4_ molecule possessing cross-orthogonal arrangement of two (As-As) bonds; (**b**)—H-saturated molecular prototype of network cluster derived from As_4_Se_2_ molecule by double breaking in Se atom positions conserving closed *tetragon*-like As_4_ arrangement of (As-As) bonds (As_4_Se_4_H_4_). The terminated H atoms are grey-colored, Se and As atoms are, respectively, blue- and red-colored, and covalent bonds between atoms are denoted by respective colored sticks. The cluster-forming energies *E_f_* are defined with respect to the energy of a single AsSe_3/2_ pyramid.

The ball-and-stick presentation in Figure 6a highlights the main features of optimized tetra-arsenic tetraselenide As_4_Se_4_ molecule [12,46]. This realgar-type molecule of *D_2d_* symmetry is composed of a maximum number of small rings (four pentagons and four hexagons) built of eight As-Se and two As-As bonds in evidently under-constrained (floppy) topology possessing *n_c_*~2.875. The *E_f_* energy for this molecule is 0.40 kcal/mol (which is dominant among all As_4_Se_4_-bearing polymorphs [46]), and intramolecular parameters are in good agreement with those refined from XRD analysis for monoclinic As_4_Se_4_ [19,20,21]. 

Since molecular clusters undergoing decomposition become As-deficient (because of the transition from coordination number CN = 2.57 corresponding to As_4_Se_3_ to CN = 2.50 in As_4_Se_4_), the appeared amorphous substance is expected to be As-enriched. Among different network clusters related to the As_4_Se_n_ thioarsenide molecules with *n_c_* < 3, only one can be derived from the under-constrained (*n_c_* = 2.67) tetra-arsenic biselenide As_4_Se_2_ molecule by double x2-breaking the Se atom positions, possessing reasonable *E_f_* energy approaching −0.72 kcal/mol. The optimized ball-and-stick presentation of the H-saturated molecular prototype of this cluster keeping a closed *tetragon*-like As_4_ arrangement of four (As-As) bonds is shown in Figure 6b. Because of such topology with only one small ring involved (As_4_ tetragon), the network built of such clusters corresponding to a-As_4_Se_2_ (with CN = 2.67) is topologically over-constrained in view of *n_c_* = 3.33.

Finally, the complete decomposition reaction in glassy arsenoselenides that are compositionally close to tetra-arsenic triselenide (g-As_4_Se_3_) can be schematically presented as follows:

2·As_4_Se_3_-I → As_4_Se_4_ + a-As_4_Se_2_   +(Δ*E_f_* = +0.41 kcal/mol). (4)

**Figure 7:**
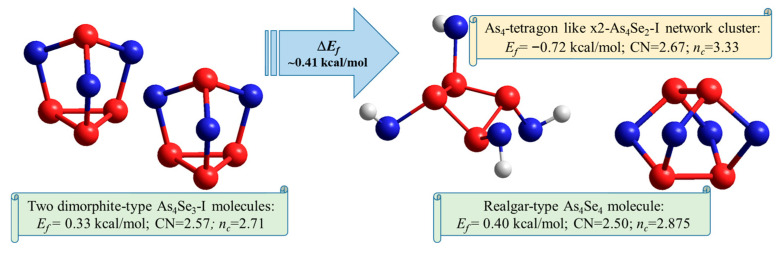
Ball-and-stick presentations of decomposition reaction changing molecular-network disproportionality in glassy arsenoselenides compositionally approaching tetra-arsenic triselenide. The two most favorable dimorphite-type As_4_Se_3_-I molecules are transformed into realgar-type As_4_Se_4_ molecules and the network-forming remainder becomes closer to amorphous a-As_4_Se_2_. The optimized configurations of molecular and network clusters are reproduced with Se and As atoms, respectively, labeled by blue- and red-colored balls, and terminated H atoms are labeled by grey balls. The decomposition barrier Δ*E_f_* derived from respective cluster-forming energies tends to ~0.41 kcal/mol.

This reaction is schematically depicted in Figure 7 using a molecular presentation of the constituents. The energetic barrier of this decomposition Δ*E_f_* is close to ~0.41 kcal/mol. It means that in contrast to the glassy-crystalline g/c-As_4_S_3_, where such reaction occurs under the barrier Δ*E_f_* ~1.5 kcal/mol [44,45], the decomposition of the As_4_Se_3_ molecular phase becomes more plausible. As a result, thermally activated crystallization in As-Se alloys is merely inhibited by the amorphous phase (a-As_2_Se_4_) which appears to extend their glass-forming ability to more As-rich compositions approaching As_70_Se_30_ [5,6,7].

By analogy with As-S alloys [44,45], the decomposition reaction (4) can be initiated by breaking one of three (As-Se) bonds within the AsSe_3_ pyramid of the most plausible dimorphite-type As_4_Se_3_-I molecule (see Figure 7). This results in a local disturbance due to the release of one Se atom from this molecule. Interacting with another such molecule, this disturbance is stabilized by forming an As-deficient with respect to As_4_Se_3_ stoichiometry (CN = 2.57) with a realgar-type As_4_Se_4_ molecule (under-constrained in view of *n_c_* = 2.875 (CN = 2.50) and the over-constrained As-enriched remainder a-As_4_Se_2_ (with *n_c_* = 3.33, CN = 2.67) is left as the destroyed As_4_Se_3_-I molecule. Specifically, the cluster contributing to such an amorphization scenario can be considered as a network-forming derivative reconstructed from the As_4_Se_2_ molecule by double x2-breaking in Se atom positions (x2-As_4_Se_2_-I cluster shown in Figure 6b).

As an alternative to this reamorphization scenario (4), the decomposition of As_4_Se_3_-I molecules into a realgar-type As_4_Se_4_ phase supplemented by a more As-enriched substance, such as tetra-arsenic monoselenide (As_4_Se) or arsenic (As_4_), can be considered. The latter is undoubtedly more essential for the above reaction (4), while not equally competitive to this decomposition reaction in view of under-estimated cluster-forming energies *E_f_* beyond the glass-forming region in binary As-Se system [5,6,7].

**Figure 8:**
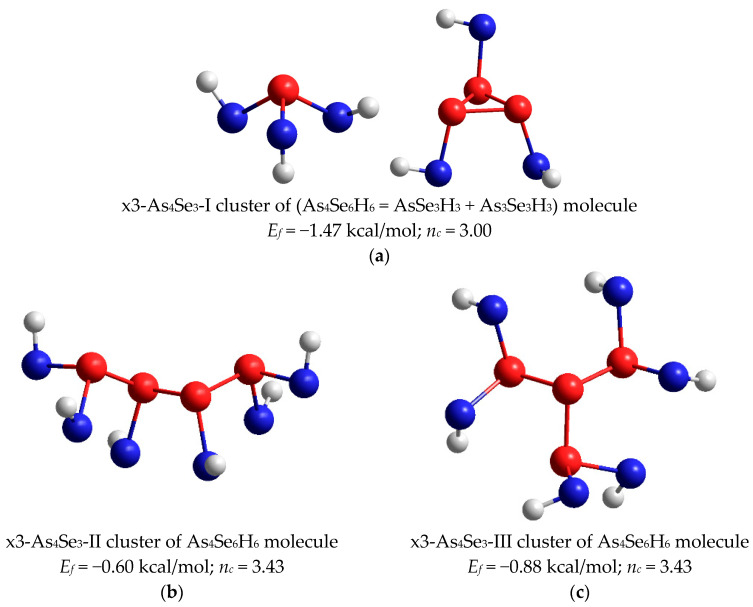
The optimized ball-and-stick presentation of most favorable molecular prototypes derived from As_4_Se_3_ molecules by breaking in Se positions, which conserve triangular-pyramidal (As_3_)-As (**a**); chain-like As_4_ (**b**) and star-like As(As)_3_ (**c**) configurations. The terminated H atoms are grey-colored, Se and As atoms are blue- and red-colored, and covalent bonds between atoms are denoted by respective colored sticks. The cluster-forming energies *E_f_* are defined with respect to the energy of a single trigonal AsSe_3/2_ pyramid.

Under high-energy MM, products of molecular-to-network transition in As-Se glass alloys can be also stabilized as the most favorable network conformations derived by direct destruction from all possible As_4_Se_3_ thioarsenide molecules. These conformations include network clusters reconstructed from these molecules by breaking in all Se atom positions (Figure 5), which appear due to an extremely large portion of mechanically transferred energy. By CINCA modeling, it was found that triple-broken derivatives of each molecule possessed better cluster-forming energy and therefore could be considered as possible in the MQ-derived alloys subjected to nanomilling. The H-saturated molecular prototypes of these network clusters conserving *triangular-*, *chain-* and *star*-like arrangement of neighboring As-As bonds are, respectively, reproduced in Figure 8a–c. 

In realistic arsenoselenide glass structures composed of the most favorable As_4_Se_3_-I molecules, as shown in Figure 5a (*E_f_* = 0.33 kcal/mol), the network clusters are possible in a reverse sequence to energetic barriers Δ*E_f_* calculated with respect to the cluster-forming energies *E_f_* provided in the captions in Figure 8. The most plausible are *chain*-like (Δ*E_f_* = 0.93 kcal/mol) and *star*-like (Δ*E_f_* = 1.21 kcal/mol) clusters. Because of the absence of small rings, both clusters are over-constrained (*n_c_* = 3.43; CN = 2.57), counterbalancing the effect from molecular dimorphite-type As_4_Se_3_-I (*n_c_* = 2.71; CN = 2.57) and realgar-type As_4_Se_4_ (*n_c_* = 2.875; CN = 2.50) clusters. Noteworthy, the optimally constrained network clusters derived from As_4_Se_3_-I molecule by triple x3-breaking in all Se atom positions (*n_c_* = 3.00), keeping basal (As_3_) triangle as a small ring (see Figure 7, are rather impossible in realistic As-Se structures because of very unfavorable cluster-forming energy *E_f_*~−1.47 kcal/mol, resulting in an unrealistically high energetic barrier of direct destruction (Δ*E_f_* = 1.80 kcal/mol). 

## 3. Materials and Methods

### 3.1. Glass Samples Preparation, Nanomilling Treatment and Preliminary Characterization

Glass samples of tetra-arsenic triselenide g-As_4_Se_3_ (*viz*. g-As_57_Se_43_) and some alloys slightly deviated from this composition (g-As_55_Se_45_ and g-As_60_Se_40_) were obtained by vibrational MQ from elemental precursors (the As and Se of 5N purity stored in argon atmosphere) [8,9,12]. 

The sealed ampoules filled with As and Se were placed in a rocking furnace, heated to 650 °C and homogenized for 10 h. Then, they were placed vertically, cooled to 500 °C for 1.5 h and quenched in water. To eliminate residual stress possible under cooling, the ingots were annealed for 1 h at 125 °C, which was below the glass-transition temperature of the samples defined from calorimetric scanning under 10 K/min heating rate (*T_g_*~140 °C for g-As_57_Se_43_). The ingots extracted from the ampoules were completely amorphous, as observed from their XRPD patterns, showing diffuse peak-halos typical for amorphous substances, conchoidal fracture and IR transparency of fresh cut-sections.

The macroscopic density *ρ* (±0.005g·cm^−3^) of g-As_4_Se_3_ defined by the Archimedes displacement method in ethanol was 4.447 g·cm^−3^, this being in good agreement with the known counterparts [5,6,7]. The mean inter-atomic spacing for this glass *d_s_^m^*~3.80 Å was the maximum among all over-stoichiometric As-rich glassy arsenoselenides g-As_x_Se_100−x_ (40 < x < 65) [9]. 

The nanomilling treatment in the high-energy planetary ball mill Pulverisette 6 (Fritsch, Germany) was employed to transform coarse-grained pieces of the prepared glasses (~3 g) sieved under 200 μm into a fine-grained (nanostructured) state. Mechanical attrition was performed in a mill for 60 min under a protective Ar atmosphere with 500 min^−1^ rotational speed in 250 mL tungsten carbide chamber loaded with 50 tungsten carbide balls (each having 10 mm in diameter). Under such MM conditions, the energy transfer to the powder estimated through specific grinding work performed in a rotational mill of this type was as high as ~300–320 kJ/g [47,48,49], justifying a novel branch of contemporary materials science and engineering, the chalcogenide mechanochemistry [2,50]. The particle size distribution was recognized for nanosuspentions prepared on the basis of powdered alloys, employing photon cross-correlation spectroscopy with Nanophox particle size analyzer (Sympatec, Germany). The unimodal particle size distribution was monitored for examined samples, showing the averaged (x50) parameter approaching ~180 nm (meaning that 50% of particles were smaller than 180 nm) and (x99) parameter close to ~330 nm (meaning that 99% of the particles were smaller than 330 nm).

### 3.2. Medium-Range Structural Research in Molecular-Network Glassy Arsenoselenides

**Figure 9:**
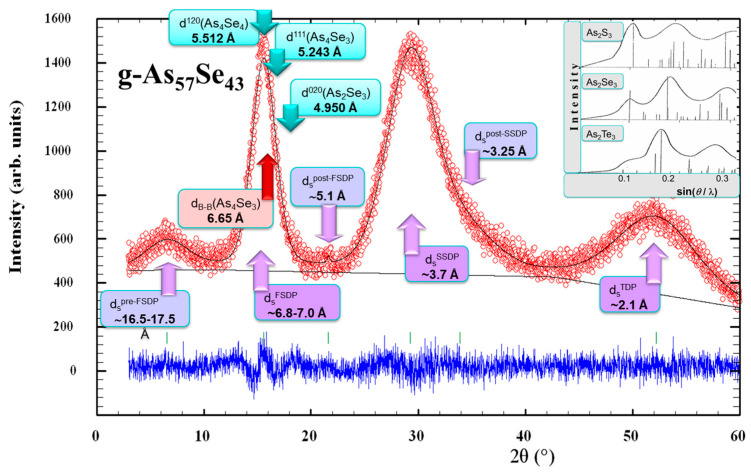
Positioning of experimental (red points) and calculated (black line) XRPD profiles in the MQ-derived g-As_57_Se_43_ showing diffuse peak-halos arrangement with respect to the characteristic inter-planar and inter-atomic (inter-molecular) correlations from quasi-crystalline arsenoselenide remnants (the difference is depicted by the blue curve at the bottom). The insert shows the comparison of “amorphous” halos and most prominent “crystalline” peaks in vitreous As_2_S_3_, As_2_Se_3_ and As_2_Te_3_ (from the *top* to the *bottom*) modified from the known work of Vaipolin and Porai-Koshits, 1963 [53].

Medium-range structure of glassy arsenoselenides was recognized with the XRPD analysis using STOE STADI P diffractometer operational in transmission mode with Cu Kα_1_-radiation and curved Ge monochromator on primary beam (more details in [8,9]). The XRPD patterns were collected under 0.015°2*θ* step, detector increment of 0.480°2*θ* and 500s scanning time per step in the whole range of angles (2*θ*). The amorphous phase was identified parameterizing diffuse peak-halos in the XRPD patterns of the examined substances (Figure 9), in part, the FSDP, serving as a signature of structural entities forming a so-called *intermediate-range ordering* (IRO) in a glass over a few tens Å reproduced in a reciprocal space near scattering vector *Q*_1_~1–1.5 Ǻ^−1^ [51], and the SSDP (according to Elliott [52]) or the PDP (the principal diffraction peak according to Zeidler and Salmon [18]) serving as the signature of *extended-range ordering* (ERO) near *Q*_2_~1.8–2.2 Ǻ^−1^. 

Specifically, in the XRPD patterns of over-stoichiometric g-As_x_Se_100−x_ (*x* > 40), the FSDP related peak-halo at ~15–22°2*θ* reflects IRO, corresponding to predominant correlations between some polyhedrons such as thioarsenide As_4_Se_n_ molecules, while the SSDP shifted to higher diffraction angles of ~28–33°2*θ* reflects ERO, corresponding to orientational arrangement of these polyhedrons (related to the second-order pair atomic correlations close to mean inter-atomic spacing in a glass, *d_s_^m^* [24]). At ~50–60°2*θ* (*viz*. *Q*_3_~3.3–4.0 Ǻ^−1^), the third peak-halo (not so sharp as the previous) known as the TDP (the third diffraction peak) is observed in XRPD patterns as a manifestation of *the shortest interatomic separation* in a glass related to the nearest-neighbor As-Se and As-As distances of ~2.1–2.3 Ǻ [54,55].

Thus, the XRPD measurements reveal the *three-peak structure* of the collected diffraction patterns as shown in Figure 9, which reflects a succession of single pairwise correlations defined presumably by *Q*_3_ = *Q*_TDP_, and multi-pairwise correlations defined by *Q*_1_ = *Q*_FSDP_ and *Q*_2_ = *Q*_SSDP_ responsible for medium-range ordering [18].

Equilibrium phase diagram of binary As-Se alloys derived with thermodynamic optimization [56] contains three stable ambient-temperature compounds, such as stoichiometric arsenic triselenide As_2_Se_3_ (corresponding to tetra-arsenic hexaselenide As_4_Se_6_) and two over-stoichiometric selenides, tetra-arsenic tetraselenide As_4_Se_4_ (arsenic monoselenide AsSe) and tetra-arsenic triselenide As_4_Se_3_, the latter existing in two modifications nominated in somewhat nonfusing nomenclature of Bastow and Whitfield [13] as high-temperature orthorhombic α-As_4_Se_3_ and ambient-temperature monoclinic β-As_4_Se_3_ phase [13,14]. Blachnik and Wickel [15] renamed β-As_4_Se_3_ and α-As_4_Se_3_ polymorphs of Bastow and Whitfield [13] in normal crystalline α-As_4_Se_3_ and α′-As_4_Se_3_ modifications stable at ambient and high temperatures, and introduced “β” symbol for plastically crystalline modification appeared under heating. As was reported by Blachnik and Wickel [15], under heating above 412 K (with 1.25 K·min^−1^ rate), the ambient-temperature normal crystalline As_4_Se_3_ modification (monoclinic α-As_4_Se_3_) transforms in high-temperature modification (orthorhombic α′-As_4_Se_3_), and under further heating above 447 K, the latter transforms into plastically crystalline β-As_4_Se_3_ phase and unidentified amorphous substance, while only orthorhombic α′-As_4_Se_3_ phase could be obtained in metastable form at ambient temperature by quenching. 

Since the examined arsenoselenides are matched within polymorphic α–α′–β phase transitions, the remainders of As_4_Se_3_ crystalline phases are expected in these alloys under nanostructurization. Hence, preliminary processing of the XRPD patterns was performed using the databases [57,58] and available resources on the crystallography of As-Se polymorphs of close compositions, in part, the JCPDS cards No. 65-2365 for monoclinic As_2_Se_3_ (space group *P*2_1_/*n*, structure type α-As_2_S_3_, orpiment [19,22]); No. 71-0388 for monoclinic As_4_Se_4_ (space group *P*2_1_/*n*, structure type α-As_4_S_4_, realgar [19,20,21]); No. 04-4979 for orthorhombic As_4_Se_3_ (space group *Pnma*, structure type α-As_4_S_3_, dimorphite [13]), and terminated elemental constituents in As-Se system, in part, the JCPDS cards No. 73-0465 for trigonal t-Se (space group *P*3121 [59]), and No. 72-1048 for rhombohedral (grey) As (space group $R\bar{3}m$ [60,61]). To visualize crystallographic details of the above phases, well known programs, such as DIAMOND [62] and VESTA [63], were employed. 

The arrangement of diffuse peak-halos in collected XRPD patterns responsible for amorphous phase was analyzed using the STOE WinXPOW 3.03 [64] and PowderCell 2.4 [65] program packages, following normalization procedure with respect to the maximum peak, which in the case of selenide ChG was related to the SSDP (in full harmony with the famous research of Vaipolin and Porai-Koshits from 1963 [53], see also insert in Figure 9). The accuracy in the peak-halo position (2*θ*) and full width at half maximum (FWHM) was no worse than ±0.05°2*θ*, the scattering vector *Q* = (4π/*λ*)·sin*θ*, and width Δ*Q* = (4π/*λ*)·sin(FWHM/2) corresponding to the peak-halo were calculated using the Bragg diffraction formalism (see, e.g., [8,9,12]). The characteristic distance *R* defined as spacing of quasi-periodicity responsible for diffuse peak-halo and correlation length *L* over which this quasi-periodicity was maintained in real space were defined as *R* = 2π/*Q* and *L* = 2π/Δ*Q*. The peak-halos in the XRPD patterns were also treated as arising from the diffraction of coordination spheres, i.e., closest inter-atomic distances like in randomly packed multiparticulate systems [24,25,26], when XRPD patterning is governed by the Ehrenfest relation [27]:

2*d_s_*·sin*θ* = 1.23·λ, (5)

where *d_s_* is the *average inter-atomic distance* between scatterers (radius of coordination sphere). Note that a realistic error bar in the above parameters (*R*, *L*, *d_s_*) does not exceed ±0.1 Å.

### 3.3. Complementary Microstructural Research on Glassy Arsenoselenides

Calorimetric heat-transfer measurements of non-reversing heat flow *HF*_nrev_ in the glasses were performed using possibilities of conventional heat-flux differential scanning calorimetry (hf-DSC) in a dynamic heating regime [34,35,66,67,68]. The instrument used for this analysis was the model DSC-1 calorimeter (Mettler-Toledo, Switzerland) equipped with a TC100 Huber intracooler. Temperature and heat calibration of the instrument was performed using a set of standard probes (water, In and Zn). For mass determination, the analytic balance model Ohaus AP250D with 0.01 mg resolution was used. The sample was encapsulated in sealed 20-μL Al pans in a N_2_ atmosphere and scanned at 10 K·min^−1^ rate. 

The nature of nanomilling-driven structural changes in glassy arsenoselenides was identified by micro-RS spectroscopy using the Horiba Xplora spectrometer equipped with a CCD detector operational at room temperature [49]. The CW 785 nm laser of 90 mW power was used for excitation, the 10% power option being used to avoid photostructural effects. Other operational options were as follows: 100× objective, 1800 mm^−1^ grating, 500 μm hole and 50 μm slit. The spectral resolution was maintained at the 2 cm^−1^ level and spatial resolution was near 2 μm. Numerous scans were performed on the sample’s surface to be sure that the micro-RS-spectra processed with Horiba LabSpec 6 software were identical. The nanomilled and unmilled glasses were compared through normalization by matching spectral areas in the region of interest. The RS-active bands in the examined samples were identified using available data for ChG analogs [35,36,37,38,39,40].

### 3.4. Cluster Modeling of Molecular-Network Conformations in As-Se Compounds

The optimized configurations of As_4_Se_3_ cage-like molecule and network derivatives were reconstructed by breaking this molecule into separate fragments linked with surrounding by Se_1/2_…Se_1/2_ bridges were reconstructed using ab initio quantum-chemical atomic cluster-modeling code CINCA [16,17]. The HyperChem Release 7.5 program based on the restricted Hartree-Fock self-consistent field method with split-valence double-zeta basis set and single polarization function 6–311G* [69,70,71] was used. Geometrical optimization and single-point energy calculations were performed by the Fletcher-Reeves conjugate gradient method until the root-mean-square gradient of 0.1 kcal/(Å·mol) was reached. The cluster-forming energy (*E_f_*) was corrected on the energy of terminated H atoms transforming the network-forming cluster in molecular one according to the known procedure [71,72], and recalculated with respect to the energy of AsSe_3/2_ pyramid (*E_f_* = −72.309 kcal/mol [17]). This modeling route (CINCA) [16,17] allows the characterization of both molecular and network configurations in saturated covalent-bonded systems like ChG [5,6], characterized by different coordination numbers (CN), parameterizing the most energetically favorable scenarios. To compare atomic clusters accounting for small rings in molecular thioarsenides As_4_Se_n_, the average number of Lagrangian constraints per atom *n_c_* was calculated for different fragments derived by breaking in available positions of Se atoms (followed by H atoms saturation), using the Phillips-Thorpe constraint-counting algorithm with stretching and bending forces ascribed to intra-molecular bonds within the cluster [73,74,75].

## 4. Conclusions

Polyamorphic (amorphous-I-to-amorphous-II) transformations driven by high-energy mechanical milling (*nanomilling*) are recognized in a melt-quenched glassy alloy of tetra-arsenic triselenide As_4_Se_3_ employing a multiexperimental approach based on the X-ray powder diffraction (XRPD) analysis of diffuse peak-halos responsible for medium-range structure ordering in glassy chalcogenides, complemented with calorimetric heat-transfer and micro-Raman spectroscopy studies.

A straightforward interpretation of medium-range structure ordering response on nanomilling-driven reamorphization in this glassy alloy is developed within a modified microcrystalline model, treating diffuse peak-halos in the XRPD patterning of chalcogenide glasses as a superposition of the Bragg-diffraction contribution from inter-planar correlations supplemented by the Ehrenfest-diffraction contribution from prominent inter-atomic and/or inter-molecular correlations belonging to some derivatives of As_4_Se_n_ thioarsenide molecules dominated by dimorphite-type As_4_Se_3_ ones. These cage-like molecules are merely destroyed under nanomilling, facilitating the formation of a polymerized covalent glass network with enhanced calorimetric heat-transfer responses. Disruption of intermediate-range ordering due to weakening of the first sharp diffraction peak (when the FSDP loses intensity, becomes broadened and shifted to higher diffraction angles), accompanied by an enhancement of extended-range ordering due to fragmentation of structural entities responsible for the second sharp diffraction peak (when the SSDP becomes broadened and shifted towards higher angles), an interplay takes place between the respective levels of medium-range structure in molecular-network As_4_Se_3_ glass undergoing reamorphization. The micro-Raman scattering spectra testify in favor of nanomilling-driven destruction of thioarsenide As_4_Se_n_ molecules and incorporation of their derivatives in a more polymerized network. The microstructure scenarios of molecular-to-network disproportionality originating from decomposition and direct destruction of As_4_Se_3_ cage-like molecules are recognized by ab initio quantum-chemical modeling employing the cluster-simulation algorithm (CINCA). 

From the point of view of the predominant microstructure, the nanomilling-driven amorphous-I-to-amorphous-II transition in glassy arsenoselenide alloys compositionally approaching tetra-arsenic triselenide As_4_Se_3_ is classified as the molecular-to-network reamorphization transition.

## Data Availability

The data presented in this study are available on request from the corresponding author.
